# *Hope therapy*: Can it treat hopelessness and internal locus of control on diabetes mellitus patients?

**DOI:** 10.1371/journal.pone.0286418

**Published:** 2023-06-13

**Authors:** Tulus Winarsunu, Lintang Aulia Utami, Siti Suminarti Fasikhah, Zainul Anwar

**Affiliations:** Department of Psychology, University of Muhammadiyah Malang, Malang, Indonesia; Dilla University, ETHIOPIA

## Abstract

Patients with diabetes mellitus (DM) are always required to be able to control a healthy lifestyle throughout their life to avoid various diseases that can accompany the previous illness. However, psychological factors in the form of despair due to lack of hope make people with diabetes more depressed and less able to control behavior and maintain blood sugar stability, so an excellent internal locus of control is needed to be stronger. This study aimed to determine the effect of hope therapy in reducing hopelessness and increasing internal locus of control in people with DM. The research design used a experimental study with ten randomly selected respondents divided into two groups, namely the control group and the experimental group. Data retrieval using the locus of control scale and the beck hopelessness scale. Data analysis used non-parametric analysis, namely the Mann Whitney test, Wilcoxon test, and Spearman’s Rank Correlation test. The results of the Mann-Whitney U test on the internal locus of control variable show a value of 0.000 and a p score of 0.008 (p <0.05), it can be concluded that there are differences in the internal locus of control in the experimental group compared to the internal locus of control in the control group. The hopelessness variable shows a value of 0.000 and a p score of 0.008 (p <0.05), this indicates that there is a difference in hopelessness in the experimental group and the control group. There is a decrease in hopelessness and an increase in internal locus of control in people with DM given hope therapy.

## Introduction

Health is everyone’s need, both physical and mental health; with a person’s mental health, other aspects of life will work optimally [1, 2]. One of the diseases that also has an impact on mental health is Diabetes Mellitus (DM) because people with DM are always required to control their lifestyle for life to avoid various risks of psychological disorders [1, 3]. DM is a disease that has blood sugar (glucose) levels that exceed normal limits, <100 mg/dl. Increased glucose in the blood due to factors that inhibit insulin action or decrease the amount of insulin. Insulin is needed so that glucose can enter the body’s cells and the glucose is then used as an energy source. For this reason, if insulin is not available or the amount is inadequate, glucose cannot enter the cells and remains in the blood in large quantities [4].

The high number of people with DM is mostly caused by lifestyle changes, such as lack of physical activity, incorrect eating patterns, poor sleep habits, high levels of stress, negative habits, and difficulty adapting to the environment [5, 6].

People with DM must constantly control their lifestyle for life to avoid various risks of psychological. Some research results show that people with DM experience psychological problems [6–8]. Patients with DM should be able to build an internal locus of control to follow the rules and change their lifestyle to support the treatment that has been done [9].

One of the important factors for controlling lifestyle is locus of control which is an individual’s belief in finding the power that controls life, especially internal locus of control where the power that controls his life comes from the individual himself [10].

Previous research has shown that several things can improve the internal health locus of control on compliance behavior and self-care behavior of patients with DM, as well as on dietary compliance and adherence to the rules of life for people with DM [9, 11]. Efforts to treat people with DM must be aware that internal factors are more important than external factors. The self-awareness that an internal factor primarily determines everything that happens is called the internal locus of control [12–14].

Individuals with an external locus of control that relate their health progress and decline due to fate or luck will tend to feel that their efforts are not producing results. In contrast, individuals with an internal locus of control are more willing to follow advice and input to treat their disease because they believe that their disease progresses depends on the management of oneself [15, 16]. The internal locus of control can be a support to help treat DM patients and provide an understanding of the psychosocial factors involved and the management of the difficulties and barriers of this chronic disease [14, 16]. Locus of control also provides positive support for DM patients to control their DM [17, 18].

Internal locus of control is very important and needed for dm sufferers because it can control behavior to obey the rules so as to improve the quality of life and health [19, 20], unfortunately, most people with DM experience anxiety, depression and even suicide [21–25], and one of the symptoms of depression is hopelessness. Helplessness is a diabetic fatalism that is owned by people with DM [26, 27]. The lack of an internal locus of control in people with DM makes them unable to control their behavior properly [16, 18].

One individual factor that does not have an internal locus of control is a sense of hopelessness; if hopelessness in people with DM can be reduced, it can increase the internal locus of control. The relationship between the internal locus of control and hopelessness is significant. However, the association between the internal locus of control and hopelessness and the discussion of the relationship between the two is still minimal. [28, 29], so it is very important to study in more depth.

Individuals who have low expectations tend to benefit the most from an expectation intervention [30–32]. Hope therapy is proven to reduce depression in patients with DM and increase hope in patients with DM [33, 34]. Several previous studies also explained that several things could reduce hopelessness, one of which is positive psychological interventions that are effective in overcoming depression, where hopelessness is the most significant factor [35, 36]. Positive psychology has a positive impact in increasing internal locus of control [37].

Hope is the most important factor for people with DM, because with the hope that people with DM can balance the psychological or physical pressures of the disease they suffer [38]. Concerning the relevant stimulus regarding the impact of DM, the patient has a sense of hopelessness, so the treatment that will be used to reduce hopelessness in patients with DM is to increase the internal locus of control by using positive psychology-based interventions, namely, hope therapy.

This study aimed to determine the effect of hope therapy on reducing hopelessness and increasing internal locus of control in people with DM. This research is expected to provide a theoretical contribution to psychology, especially regarding the internal locus of control and hopelessness in people with DM. The benefits of research can help people with DM to reduce feelings of hopelessness and increase internal locus of control to keep patients away from the prolonged negative impact of their disease. Health workers and related people with DM can be used as helpful information during the treatment process.

## Theoretical analysis and hypothesis

### Internal locus of control

Locus of control was invented by Rotter in the 1960s [39], Locus of control refers to the individual’s belief in controlling his life and assessing what happens in his life; locus of control is divided into two main parts, namely the internal locus of control is an individual who believes that whatever happens in his life is his responsibility and holds his control over the events that occur on his life. Meanwhile, the external locus of control believes that individuals do not control their lives and depend on external factors. They see that external factors are responsible for what happens to them, such as luck, fate, and opportunity [37, 39].

The cause of the internal or external locus of control is due to the experience related to the failure he experienced, and this factor can sometimes be confused regarding the behavior given to the experience. Some people can have different interpretations of reinforcement and rewards offered by others [40–43].

The concept of locus of control is defined as an individual’s belief in locating the forces that control life. Internal locus of control is defined as the individual views the power that controls his life that comes from the individual himself in being responsible for what happens during his life [10]. Individuals with an internal locus of control believe that their actions and behavior determine whatever happens to them, including their health [44]. Individuals with an internal locus of control perspective assess any event as non-threatening and feel challenged to deal with it, which is associated with improved healthy behavior [44, 45].

### Hopelessness

Negative and repetitive life events can contribute to the formation of hopelessness because it will lead gradually to the belief that there is powerlessness in the face of stressors. Individuals who respond to negative events form three dimensions of causal attribution, namely internal to external, stable to unstable, and global to specific [47]. The development of the causes of hopelessness leads to a negative inferential style which is divided into three, 1) referring to the tendency to attribute negative events, 2) the tendency to perceive negative events as having many negative consequences, and 3) the tendency to infer negative characteristics about oneself when negative events occurred [46, 47].

Hopelessness theory describes that the three negative inferential forces function as a cognitive diathesis that cannot be associated with the possibility of increasing hopelessness or depression. On the other hand, when faced with negative events, only individuals with cognitive diathesis are at greater risk of hopelessness and depression because this hopelessness theory is a stress diathesis depression model. In summary, individuals with cognitive diathesis should not be at risk for hopelessness or depression in the presence of positive events or the absence of negative events [48–50].

Hopelessness is understood as a core element of negative expectations about an unappreciated outcome or feeling powerless to change the likelihood of that outcome [52]. Hopelessness is understood as a core element of negative expectations about an unappreciated outcome or feeling powerless to change the likelihood of that outcome [51].

### Hope therapy

Hope therapy states that a person’s emotions follow the desired goal; when thoughts are directed to a positive realm, emotions will reflect as they think. People with low expectations tend to think negatively about their desires, impacting their confidence and ability to achieve their dreams [51, 52].

Hope is a force that can indeed function as an essential therapy for change by taking advantage of current expectations. Some of the principles that support hope therapy, including the fact that hope therapy is designed to be a short and semi-structured form of treatment in Indonesia whose primary focus is the current target [53].

In addition, to increase hope, the therapist helps clients to focus on goals, possibilities, and past successes rather than on problems or failures. There are four main components in hope therapy, namely, Finding Hope, Bonding Hope, Raising Hope, and Reminding Hope [54].

The components in hope therapy are divided into four; namely, Hope Finding is a search for hope and is owned by every client to be built and developed as a therapeutic process. Every individual has a different way of wishing, and various expectations can be in the form or purpose [55]. The second component is the Bonding Hope, in which the therapist and client build a solid and hopeful collaboration with the client to achieve what they have been looking for. Building positive and therapeutic energy for clients by working together is the key. The third component is Raising Hope, assisting clients in increasing hopeful thinking in general in the life they want to improve. The last component is Reminding Hope by teaching them how to monitor themselves and use techniques to support themselves independently when separated from therapy [35, 56].

### Hope therapy to reduce despair and improve internal locus of control

People with DM tend to experience symptoms of depression due to their illness; one of the symptoms of depression that is the main trigger is hopelessness. Despair makes it difficult for people with DM to internalize their disease to help their recovery. People with DM who experience hopelessness tend to have an external locus of control. Hence, people with DM feel that their efforts to recover are futile because they put their hopes and actions on the outside [57–59]. In despair, there is an aspect of tendency to attribute negative events. Then the tendency to perceive negative events as having many negative consequences and to infer negative characteristics about oneself when negative events occur. Individuals can balance their thoughts regarding negative stressors in their lives to have an internal locus of control [60–62].

The general expectation that the outcome of an event depends on the individual’s abilities and efforts is called the internal locus of control [10] and identification of hopelessness is a major characteristic of a pathological state consistent with a lack of purpose and meaning in life in depressed individuals. As a result locus of control is positively related to hopelessness. More specifically, individuals with an external locus of control are more likely to experience hopelessness and depression [61, 62]. The relationship of hopelessness with locus of control causes a person to believe that he has no control over the occurrence of conflict events and losing hope [63–66].

Thus it can be concluded that when hopelessness decreases, the internal locus of control will increase, to reduce despair, hope therapy is given as a form of positive psychological intervention that focuses on improving hope.

### Hypothesis

Hopelessness decreases through hope therapy and internal locus of control increases in people with diabetes mellitus.

## Methods

Research proposals are presented before the ethics committee to ensure the impact of the research to be carried out, while ensuring that it does not violate the code of ethics. The results of the study by the Experimental Research Ethics Commission of the Faculty of Psychology, University of Muhammadiyah Malang, approved the research (research ethics approval number: E.6.m/415/FPsi-UMM/XII/2021). In addition, each participant is also required to provide willingness and commitment (informed consent) during the intervention process. The authors assured the participants that participant data would be kept confidential.

### Research design

This study uses a experimental type of research with a control group experimental design pre-test-posttest design; in this design, there are two groups selected randomly, then given a pretest to determine the initial state of the experimental group and the control group. After being manipulated, the dependent variable was re-measured with the same measuring instrument in two groups with two different situations.

### Research subject

The participants in this study were people with DM in the Bareng Village area of Malang City Indonesia as many as 57 people, but after screening and asking for willingness, only 10 people were willing to be participants so that the ten people with DM were divided into two groups, five people as an experimental group and five people as a randomly selected control group. Participants between the ages of 30–50.

### Research instruments

Hopelessness was measured using the Beck Hopelessness scale (BHS) which was adapted from research by Sarfika [67] as many as 20 items with a reliability of 0.766. Each item is answered by choosing true or false according to the participant’s condition. Internal locus of control was measured using a locus of control scale adapted from research Nisa [68] as many as 20 items with a reliability of 0.77. Each item is answered by choosing true or false and is rated 0 or 5, making this test score ranges from 0 to 100; the higher the individual score, the stronger the internal locus of control.

### Research procedures and data analysis

The research procedure begins with a literature review. It determines research methods that are relevant to the research topic, followed by searching for respondents who are following the characteristics of research respondents, one of the characteristics is that respondents are willing and committed to participating in the program as well as internal locus of control scores and the Beck hopelessness scale in the low category. (pre-test data). Ten respondents matched the characteristics of the research subjects; then, a random assignment process was carried out to determine the experimental and control groups. The experimental group was given intervention or treatment in the form of hope therapy.

Hope therapy is carried out in 12 intervention sessions for 30–45 minutes, which consists of; 1) Hope finding (1 session), that is, providing knowledge about expectations and finding out and exploring the desires and expectations of the subject based on aspects, 2) Hope bonding (2 sessions), that is, fostering a strong and hopeful alliance or cooperation with the subject, 3) Hope enchancing (4 sessions), that is, increasing hopeful thinking on the subject and providing feedback related to the subject’s thoughts regarding expectations, 4) Hope reminding (3 sessions), that is, teaching the subject how to monitor himself by using their thoughts about the expectations that have been made before and making plans after the therapy has been completed, 5) Termination (2 sessions), i.e. closing the intervention by drawing conclusions from the intervention sessions that have been carried out, looking at the difference between the initial condition and the last condition after the intervention is carried out and the administration of posttests using the internal locus of control and beck hopelessness scale [54].

The implementation of the intervention, in the form of hope therapy, was carried out in the experimental group. No treatment was carried out in the control group by the intervention module that had been prepared and tested. After the intervention process was carried out, measurements were taken (post-test data) to see the effect of hope therapy on hopelessness and internal locus of control.

After the intervention process is complete and the data is collected, data analysis is carried out using non-parametric data from the Mann-Whitney and Wilcoxon Test. Mann Whitney test to test the hypothesis; if the significance value > 0.05, then H0 is accepted, and Ha is rejected. If the significance value is <0.05, then H0 is rejected, and Ha is accepted. Meanwhile, Wilcoxon to determine the difference between the results of the pretest and post-test in both control and experimental groups or to test the effectiveness of the therapy with the help of the SPSS version 23 for windows program. In addition to quantitative data, interviews were conducted before and after treatment to determine differences in psychological conditions that could not be obtained through a scale as additional data that was considered essential for the psychological well-being of respondents, as well as an evaluation of the intervention process.

## Results

Based on the results of the study, a description of the study subjects consisting of 10 participants (5 participants in the experimental group and 5 control groups was obtained). As for more details as in Table 1.

**Table 1:** Description of research subjects.

Subject	Sex	Age	Type DM	experimental group
1	Female	42	Type 2	experimental group
2	Female	40	Type 2	experimental group
3	Male	30	Type 2	experimental group
4	Female	50	Type 2	experimental group
5	Male	39	Type 2	experimental group
6	Male	44	Type 2	control group
7	Female	50	Type 2	control group
8	Female	30	Type 2	control group
9	Male	47	Type 2	control group
10	Male	49	Type 2	control group

The results showed that the comparison of pretest and posttest on internal locus of control variables in each group to answer the research hypothesis using the Wilcoxon test because there were too few study subjects and abnormal data, as in Table 2.

**Table 2:** Comparison of pretest scores and posttest scores of internal locus of control.

Group	Mean		Standard Deviation		Sig.
	*Pretest*	*Posttest*	*Pretest*	*Posttest*	
Experiment	35,00	60,00	7,071	7,906	0,041
Control	40,00	35,00	7,071	3,535	0,197

In Table 2, the experimental group received treatment in the form of hope therapy with an internal locus of control pretest score of (M = 35.00 with SD = 7.071). Then the post-test score obtained results of (M = 60.00 with SD = 7.906). That way, the average value of the posttest is greater than the average value of the pretest on the internal locus of control variable with the Asymp value. Sig (2-tailed) is 0.041 < 0.05, so it can be concluded that there is a significant difference in the pretest and posttest scores of the experimental group. The increase in score indicates that there is an increase in the internal locus of control in the experimental group after being given the intervention or treatment.

Table 1 shows that the control group had a pretest score on the internal locus of the control variable (M = 40.00 with SD = 7.071), then the post-test score obtained a result of (M = 35.00 with SD = 3.535). That way, the average value of the posttest is smaller than the average value of the pretest on the internal locus of control variable with the Asymp value. Sig (2-tailed) of 0.197 <0.05, so it can be concluded that there is no significant difference in pretest and posttest scores.

The Mann-Whitney test was performed on both variables to see the differences in internal locus of control and hopelessness in the experimental and control groups. The results of the Mann-Whitney U test on the internal locus of control variable show a value of 0.000 and a p score of 0.008 (p <0.05), it can be concluded that there are differences in the internal locus of control in the experimental group compared to the internal locus of control in the control group. This shows that Ha is proven, namely that there is a difference in the internal locus of control between the control and experimental groups, where the internal locus of control in the experimental group has changed scores compared to the control group. The hopelessness variable shows a value of 0.000 and a p score of 0.008 (p <0.05), this indicates that there is a difference in hopelessness in the experimental group and the control group, which supports the proof of Ha, namely the difference in despair between the two groups, namely the experimental and control groups.

Spearman Rank correlation analysis was used to determine the relationship between the two variables, namely despair and internal locus of control. It is known that the correlation coefficient of the hopelessness variable on the internal locus of control is -0.937 with a p-value of 0.000 (p<0.05), and the direction of the relationship is negative. Thus, it can be concluded that hopelessness negatively correlates with an internal locus of control. That is, the lower one’s despair, the higher the internal locus of control one has.

In addition to the results of the quantitative analysis, guided interviews were also conducted through items on the internal locus of control scale and the hopelessness scale. The following is additional data obtained through the results of interviews between before and after the treatment was given, it can be seen in Tables 3 and 4 below:

**Table 3:** Table of changes in internal locus of control before and after intervention.

Before Intervention	After Intervention
• Feeling that the effort in treatment is in vain and depends on fate or destiny	• Understand that the effort to seek treatment is one’s own decision and is one’s responsibility.
• Assuming that what determines health is not one’s responsibility	• Being able to understand the health of each individual is the responsibility of each individual
• Blaming the environment and others for what happened to themselves	• Understand that what happens to oneself is the result of one’s actions.
• Difficulty taking lessons from events in life	• Able to interpret events in life more positively.

**Table 4:** Table of changes in hopelessness before and after intervention.

Before Intervention	After Intervention
• Easily giving up and feel helpless over the disease you have	• Optimizing existing capabilities and not focusing on negative situations
• Inability to manage desires and abilities due to illness constraints	• Able to know the abilities and strengths they have even though they have a disease
• Feeling afraid of the future and have no control over life	• Understand that the disease they have can still be controlled by theirself as long as there is effort and will
• Feel lazy and think the effort is just vain	• Understanding the control they have as a patient

## Discussion

The results showed a significant increase in the internal locus of control by reducing hopelessness using hope therapy in people with DM. The experimental group that was given this intervention had lower hopelessness than the control group, so an increase in the internal locus of control occurred significantly in the experimental group. The results showed an increase in hopelessness in the control group, although the increase was not significant even though no treatment was given. This proves that the control group that is not given any treatment can increase despair if it is not handled correctly, which means it is essential to provide treatment to hopeless people.

The existence of the role of hopelessness in influencing a person’s locus of control is explained through previous research that a person’s sense of hopelessness does occur due to the influence of last negative events. Still, when the feeling of hopelessness can be controlled, it will change the control center in internalizing an event [67–69].

Despair is very vulnerable to occur in people who suffer from DM because DM is a chronic disease and lasts a lifetime, so it can affect how the sufferer views his or future [67].

Hopelessness is negative energy that a person has, especially for people with unpleasant events such as suffering from DM. When one is hopeless and has no hope, it will be difficult for a person to have a locus of control. Binzer explained that negative energy, such as loss of motivation, can contribute to a low internal locus of control and vice versa to provide control over himself and tend to depend on the circumstances around him [70].

Based on research, Hope therapy can effectively reduce hopelessness in people with DM. Changes occurred in several sessions conducted with aspects of hopelessness and the presence of other factors, namely support from family, which decreased despair in each session. In the therapeutic process, the experimental group has a higher level of hopelessness than the control group. Therefore it is essential to pay more attention to the barriers and supports for people with DM in the experimental group.

This research still has limitations in terms of time for therapy because it was carried out during the Covid-19 pandemic, resulting in researchers having limited time and space due to health protocol regulations and considering the safety of research subjects.

## Conclusion

Based on the research that has been done, it can be concluded that there is a decrease in hopelessness and an increase in internal locus of control in people with DM given hope therapy. In addition, research subjects can also understand that the illness they suffer can be controlled and is a personal responsibility that can be handled alone. The implications of this research are expected to bring awareness to people with DM to keep hope, not give up on their illness, and be able to internalize negative events in life and not surrender or surrender to fate.
